# DNA甲基化在肺癌顺铂耐药中的研究进展

**DOI:** 10.3779/j.issn.1009-3419.2023.101.03

**Published:** 2023-01-20

**Authors:** Jinzhe SUN, Jun CHEN

**Affiliations:** 116000 大连，大连医科大学附属第二医院肿瘤科; Department of Oncology, The Second Hospital of Dalian Medical University, Dalian 116000, China

**Keywords:** 肺肿瘤, DNA甲基化, 去甲基化, 顺铂, 耐药性, Lung neoplasms, DNA methylation, Demethylation, Cisplatin, Drug resistance

## Abstract

肺癌作为最常见的恶性肿瘤之一，严重威胁人类的生命健康。铂类药物如顺铂（cisplatin, DDP）是肺癌的一线治疗药物，但其获得性耐药是肺癌治疗的主要难题，也是预后不良的关键原因。顺铂耐药的发生机制较为复杂，确切机制尚未明确。大量研究表明DNA甲基化在肺癌顺铂耐药过程中发挥关键作用，DNA异常高甲基化使染色质构象发生变化，导致多种耐药基因和抑癌基因失活。阐明其复杂机制有望为寻找预测疗效的生物标志物和治疗新靶点的确定提供依据。因此，本文就DNA甲基化在肺癌顺铂耐药过程中的作用和机制做一综述，并总结近年来的去甲基化策略，为逆转肺癌顺铂耐药提供新思路。

## Abstract (en)

As one of the most common malignant tumors, lung cancer poses a serious threat to human life and health. The platinum-based drug cisplatin (DDP) is used as the first-line treatment for lung cancer. The poor prognosis of lung cancer is mostly due to developed resistance to cisplatin, which poses a serious treatment challenge. The mechanism of cisplatin resistance is complex and unclear. Numerous studies have shown that DNA methylation plays a crucial role in the emergence of lung cancer cisplatin resistance. DNA hypermethylation results in the deactivation of numerous drug resistance genes and tumor suppressor genes through a change in chromatin conformation. Finding new therapeutic targets and indicators to predict the therapeutic effect can be aided by elucidating the complex mechanism. In order to discover novel strategies to overcome cisplatin resistance in lung cancer, this paper discusses DNA methylation-mediated cisplatin resistance and offers an overview of current demethylation procedures.

在21世纪，肺癌是全球范围内发病率和死亡率最高的肿瘤性疾病^[1]^，我国肺癌发病率和死亡率也在逐年攀升^[2]^。化疗是晚期肺癌的重要治疗手段，顺铂（cisplatin, DDP）则是临床上最活跃的抗癌药物之一，含铂双药化疗是无驱动基因突变患者的主要一线治疗^[3,4]^，但顺铂耐药限制了其临床应用，成为肺癌治疗亟待解决的问题之一。因此了解顺铂耐药的分子机制、寻找预测标记物、开发更有效的药物来逆转顺铂耐药是必要的。随着甲基化检测技术的发展和表观遗传学研究的深入，学者们逐渐认识到DNA甲基化在肿瘤耐药过程中发挥着重要作用。在肺癌中常伴随着DNA异常甲基化，研究者^[5,6]^发现共有372个基因在顺铂耐药细胞株A549/DDP中表现出高甲基化，这些基因参与了最基本的生命过程，如细胞增殖、凋亡等。因此，本文重点描述DNA甲基化与肺癌DDP耐药的相关机制，综述近年来的研究热点基因，总结既往的研究成果，为进一步研究提供思路和方向。

## 1 肺癌DDP耐药机制

含铂双药化疗一直是无驱动基因突变的转移性肺癌治疗的基石^[4]^，Schiller等^[7]^对DDP联合三代化疗药物（如紫杉醇、多西他赛）在转移性非小细胞肺癌（non-small cell lung cancer, NSCLC）患者中进行研究，结果显示反应率为15%-20%，总生存期（overall survival, OS）为7个月-8个月。越来越多的证据表明，化疗的一些抗肿瘤活性可能是通过免疫介导。特别是，化疗被认为能在肿瘤细胞上诱导更多的程序性死亡配体1（programmed cell death ligand 1, PD-L1）表达，并增加肿瘤抗原的呈递^[8,9]^，这也是将含铂化疗与免疫治疗相结合的原理。因此，即使在免疫和靶向治疗的时代，以DDP为基础的化疗仍然是中晚期肺癌的一线治疗方案^[3,10]^。DDP属于细胞周期非特异性药物，具有细胞毒性。它含有类似烷化剂的双功能基团，其抗肿瘤的主要机制是作用于DNA形成链间及链内交联的DDP-DNA复合物，造成DNA损伤或干扰DNA的复制、转录和翻译，还可与核蛋白及胞浆蛋白结合，抑制RNA与蛋白质合成^[11]^。

然而，长期使用DDP不可避免会出现耐药，约30%初次及70%多次DDP化疗的患者会出现耐药^[12]^。研究^[13,14]^表明，DDP耐药是肺癌治疗失败的关键原因之一。DDP的耐药机制复杂，主要包括：（1）影响DNA损伤修复，如DNA错配修复基因（mismatch repair, MMR）缺陷引起DNA损伤耐受；（2）影响细胞内药物的积累，如通过ATP结合盒（ATP-binding cassette transporter, ABC）转运蛋白家族成员介导的DDP排泄增加和摄取减少；（3）“逃避”细胞凋亡信号，如Bcl-2蛋白家族、肿瘤抑制蛋白p53表达改变使细胞凋亡受到抑制等；（4）刺激生存信号的转导，影响与DDP诱导细胞死亡没有明显联系的分子途径，如肿瘤干细胞的形成和肿瘤微环境的改变^[15,16]^。但是，目前还没有任何一种机制能准确、完全地解释DDP的耐药性。随着探索的不断深入，越来越多的研究^[5,6]^发现在原发性耐药肿瘤中存在着耐药基因的表达异常，大多数都是由DNA甲基化异常所导致。此外，耐药模型研究^[17]^也显示，细胞毒药物产生作用后肿瘤细胞的某些耐药基因会发生DNA甲基化，促进耐药的产生。因此，DNA异常甲基化可能在肺癌DDP耐药中起非常重要的作用。

## 2 DNA甲基化

### 2.1 DNA甲基化的概念

表观遗传学是指基于非基因序列改变所致基因表达水平的变化^[18]^，DNA甲基化是表观遗传学的重要组成部分，与DNA修复、调节细胞周期和凋亡等关键过程相关。DNA甲基化是指基因组胞嘧啶-鸟嘌呤（CpG）二核苷酸在DNA甲基化转移酶家族（DNA methyltransferases, DNMTs）的催化下将S-腺苷酸甲硫氨酸（S-adenosine methionine, SAM）上的甲基转移到DNA分子上，使基因启动子区CpG二核苷酸5’端的胞嘧啶转变为5’甲基胞嘧啶的过程^[19]^。CpG二核苷酸在基因组中的分布不均匀，出现频率较高的一段区域被称为CpG岛（CpG island, CGI）^[20]^。在人类中，CGI一般存在于基因组的第1外显子区和启动子区^[21]^，具有调控基因表达的作用。在肿瘤中，尽管基因组整体低甲基化，某些基因仍会由于调控区域CGI的高甲基化而失活，而在非恶性组织中则是未甲基化的^[22]^。DNA异常高甲基化往往能够通过降低肿瘤抑制基因的转录活性继而对该基因的表达产生影响，例如表达降低或沉默，从而间接诱导肿瘤的发生和耐药。

### 2.2 DNA甲基化的检测

DNA甲基化是当今肿瘤研究的热点，其检测方法也逐步得到完善。精准破译DNA在基因组上的位置信息有助于阐明DNA甲基化的生物学功能^[23]^。最经典的是亚硫酸盐介导法^[24]^，除此之外，还有酶促甲基化测序法和TET辅助吡啶硼烷测序法等^[25]^，但上述方法较容易产生假阳性结果，存在一定误差^[26,27]^。近年来随着第三代测序技术的兴起出现了更有潜力的检测技术，如纳米孔测序可以直接对DNA甲基化进行定位，但仍处于发展阶段，需要进一步优化检测条件以较好地维持酶活性和稳定性^[28]^。在分析微量样品时可采用单分子实时测序^[29]^，该方法具有测序通量高、成本低、易于操作等优点，但仍需解决荧光信号干扰等问题。

## 3 DNA甲基化介导肺癌DDP耐药的机制

目前，在肺癌DDP耐药方面研究最为集中和深入的甲基化基因有MMR、ABC、RAS相关区域家族1A基因（Ras association domain family 1A gene, RASSF1A）和编码钙黏蛋白超家族的基因等。它们介导肺癌DDP耐药的机制不同，因此开展深入的研究对肺癌的治疗、逆转DDP耐药具有重要意义。

### 3.1 MMR

与DDP敏感细胞相比，DDP耐药细胞显示出更强大的DNA修复能力来消除DDP诱导的DNA损伤^[30]^。DNA错配修复系统是细胞复制的一种修复机制，能够特异性识别DNA复制过程中出现的碱基错配、缺失错配和插入。其存在为DNA复制的高保真性和遗传物质的完整性与稳定性提供了保障。研究^[31]^发现，MMR还参与了细胞凋亡与增殖的调控。到目前为止共发现了9个错配修复功能基因，其中hMLH1、hMSH2最为重要。研究^[32]^表明hMLH1启动子甲基化可导致体外和体内DDP耐药的发生，目前机制尚不明确。有研究者^[33]^认为当细胞hMLH1甲基化时，细胞不能有效识别DDP介导的DNA损伤，受损细胞不会停留在G_2_期，或者抑制p53/非p53凋亡途径，继续进行复制，从而表现为耐药。hMLH1甲基化会增加基因组的不稳定性，引起基因功能丧失，产生对DDP耐受的突变体。需要注意的是，尽管hMSH2也在减少基因突变和维护基因稳定中发挥重要作用，但是目前没有关于其甲基化与肺癌DDP耐药的相关性研究。

### 3.2 ABC

DDP在细胞内的积累减少是耐药的主要原因之一。ABC转运蛋白超家族是一类三磷酸腺苷（adenosine triphosphate, ATP）依赖性药物外排泵，通过减少细胞中的药物积累使肿瘤细胞对多种抗肿瘤药物产生耐药^[34]^。多药耐药相关蛋白（multi-drug resistance associated proteins, MRPs）、P-糖蛋白（P-glyeoproteinn, P-gp）等经典的多药耐药蛋白均属于ABC转运蛋白家族。MRPs在正常组织中主要分布在内膜系统上，表达量低。相反，MRPs在肺癌组织中主要位于细胞膜且表达升高^[35]^。DDP与谷胱甘肽偶联成转运复合物后被MRPs识别，通过主动转运的方式转移至细胞外，细胞内药物浓度降低进而导致耐药。因此DDP耐药与MRPs的表达有紧密的联系^[36]^。多药耐药基因1（multidrug resistance gene 1, MDR1）编码的P-gp过度表达也是化疗耐药的主要机制之一，与人肺腺癌细胞DDP耐药相关^[34]^。MDR1基因启动子低甲基化造成基因表达过度，导致肿瘤细胞产生由P-gp介导的多药耐药^[37]^。在肺癌细胞中MDR1基因和MRP启动子附近CpG含量丰富^[38]^，因此针对两者进行DNA甲基化的研究对明确及逆转肺癌DDP耐药具有重要意义。逆转MDR的策略主要集中在抑制或调控P-gp的活性，有研究^[39,40]^显示，大黄素和橙皮素可以通过抑制P-gp的表达逆转肺腺癌细胞的DDP耐药，可能与核因子（nuclear factor, NF）-κB通路相关。

### 3.3 RASSF1A

RASSF1A编码Ras效应蛋白，是一种被广泛研究的抑癌基因，具有调控细胞周期、诱导细胞凋亡等作用。RASSF1A因异常高甲基化失活最早在肺癌中被发现，其在NSCLC中的甲基化率为63%，在小细胞肺癌（small cell lung cancer, SCLC）中更是高达80%，是人类肿瘤中甲基化频率最高的基因之一^[41⇓-43]^。尽管目前没有关于RASSF1A甲基化与肺癌DDP耐药的相关研究，但有学者^[42]^发现RASSF1A的甲基化状态在A549和A549/DDP之间存在明显差异，使用去甲基化剂会明显降低A549/DDP的活力，以剂量依赖的方式诱导A549/DDP凋亡。其机制有可能是RASSF1A通过灭活Keap1-Nrf2通路激活自噬，或激活Ras通路调节细胞凋亡，增强对DDP的敏感性^[44,45]^，这提示了该基因甲基化与DDP耐药具有潜在联系。

### 3.4 钙黏蛋白超家族编码基因

CDH13基因编码T-cadherin蛋白^[46]^，同时也是一种抑癌基因，具有负性调节细胞生长的作用，影响肿瘤的发生发展。Feng等^[47]^发现，与正常肺组织相比，NSCLC组织中CDH13启动子区呈现高度甲基化^[48]^。去甲基化恢复CDH13的表达后可以逆转肺癌DDP耐药，随着甲基化抑制剂浓度的升高，CDH13的非甲基化状态增强，可加强DDP对A549/DDP的凋亡作用。CDH1编码E-cadherin蛋白，研究显示DDP耐药细胞中的甲基化频率较DDP敏感细胞明显升高^[49]^，并且DDP对CDH1阴性的SCLC分型具有良好的疗效^[50]^，但其机制尚不明确。

### 3.5 胰岛素样生长因子结合蛋白家族（insulin-like growth factor-binding protein, IGFBPs）

IGFBP-3是IGFBPs家族含量最丰富的蛋白，其基因启动子异常高甲基化与NSCLC患者的DDP获得性耐药有关，部分机制是启动子甲基化导致该基因表达丧失并激活PI3K/Akt通路^[51,52]^。有研究^[53]^分析了NSCLC标本的基因表达，发现IGFBP-3基因启动子未甲基化有延长I期患者无病生存期（disease free survival, DFS）的趋势。因此治疗前IGFBP-3的甲基化状态可能是DDP疗效的临床生物标志物和预测因子，有助于筛选从DDP治疗中受益的患者。

除了上述基因和蛋白家族外，还有一部分研究较少的基因，其甲基化也与肺癌DDP耐药相关（表1）^[54⇓⇓⇓⇓⇓-60]^。传统肺癌化疗方案的制定取决于肿瘤的分型和肿瘤原发灶-淋巴结-转移（tumor-node-metastasis, TNM）分期，但是同一病理类型的患者疗效有时相差甚远。随着研究的深入，证实了基因甲基化与表达水平的检测可以作为预测肺癌DDP耐药的生物学标志应用于临床，多个标志物联合应用可以更准确地筛选出DDP敏感患者，这些标志物甚至有望成为逆转DDP耐药的治疗靶点辅助治疗。

**表1:** 与肺癌顺铂耐药相关的DNA甲基化基因

Gene	Changes in promoters	Mechanisms causing cisplatin resistance in lung neoplasms
hMLH1^[33]^	Hypermethylation	Inhibit p53-dependent or -independent apoptosis pathway
MDR1^[37,40]^	Hypomethylation	Enhance the expression of a P-gp
RASSF1A^[44,45]^	Hypermethylation	Activate the Keap1-Nrf2 pathway to inhibit apoptosis; Inhibit the Ras signaling pathway
CDH13^[47]^	Hypermethylation	Inhibit the cisplatin-induced apoptosis
IGFBP-3^[51,52]^	Hypermethylation	Activate the PI3K/Akt signaling pathway
FOXF1^[54]^	Hypomethylation	Inhibit the cisplatin-induced apoptosis
SOX1^[55]^	Hypermethylation	Enhance the cisplatin-induced NSCLC cells apoptosis
NNT^[56]^	Hypermethylation	Reduced NAD+ levels limit autophagy pathways and inactive SIRT1
HOXA11^[57]^	Hypermethylation	Activate the Akt/β-catenin signaling pathway
SLFN11^[58,59]^	Hypermethylation	Enhance DNA repairing system; Inhibit G_1_/S arrest
PDE3A^[60]^	Hypermethylation	Inhibit the cisplatin-induced apoptosis

The methylated genes associated with cisplatin resistance in lung neoplasms in recent years are summarized and their mechanisms are briefly reviewed. MDR1: multidrug resistance gene 1; P-gp: P-glyeoproteinn; RASSF1A: Ras association domain family 1A gene; IGFBPs-3: insulin-like growth factor-binding protein-3; FOXF1: forkhead box F1; NNT: nicotinamide nucleotide transhydrogenase; PDE3A: phosphodiesterase; NSCLC: non-small cell lung cancer.

## 4 去甲基化的治疗策略

DNA甲基化是一种可逆的表观遗传学修饰方式，因此研究DNA甲基化与肺癌DDP耐药的关系有望成为逆转耐药的新方向。由于肺癌中的异常甲基化基因多表现高甲基化，因此，目前针对肺癌DNA甲基化的治疗策略集中在去甲基化上。公认的去甲基化治疗策略主要包括DNMTs抑制剂、靶向下调基因表达和联合治疗。

### 4.1 DNMTs抑制剂

在肿瘤中常检测到DNMTs高表达，因此抑制DNMTs活性可作为肿瘤治疗的新思路^[61]^。目前，5-氮-2-脱氧胞苷（5-aza-2-deoxycytidine, 5-Aza-dc）是体外最常用且作用最强的一种甲基化抑制剂，其机制是抑制DNMTs从而抑制基因甲基化^[62]^。低剂量5-Aza-dc可以部分逆转A549对DDP的耐药性，进一步发现低剂量5-Aza-dc去除了部分hMLH1高甲基化，上调了mRNA和蛋白的表达。其临床药物如地西他滨（Decitabine, DAC）已被美国食品药品监督管理局（Food and Drug Adminstration, FDA）批准用于血液系统恶性肿瘤的治疗。在关于肺癌的临床试验中也发现DNMTs抑制剂具有一定的疗效。一项关于DAC治疗肺癌的I期临床试验^[63]^显示，36%的患者中观察到靶基因诱导。此外，一些心律失常药物如普鲁卡因胺也具有去甲基化作用，虽然作用与DAC相比较差，但已通过FDA认证并可用于治疗。还有一些靶点仍处于体外细胞和动物实验阶段^[64]^，需要进一步验证。

### 4.2 靶向下调基因表达

FDA认证了几种DNA甲基化抑制剂，如DAC、Azacitidine等，但是随之而来的各种副作用也限制了它们的临床应用，靶向治疗可能最大限度降低广谱药物副作用。RNA干扰（RNA interference, RNAi）技术通过靶向降解信使RNA从而选择性地抑制基因表达^[65]^，外源性引入小干扰RNA（small interfering RNA, siRNA）可诱导RNAi的发生，该过程遵循碱基互补配对原则^[66]^。该项技术沉默DNMTs和一些DNA启动子区基因表达也能达到逆转耐药的效果。反义寡核苷酸是一类人工合成的反义表达载体的寡核苷酸片段，导入细胞或个体后通过序列特异地与靶基因DNA或mRNA结合而抑制该基因表达。这两者均为基因沉默技术，可以改变DNA甲基化状态，逆转肿瘤耐药。现有一项正在进行的I期/IB期研究，在NSCLC等肿瘤患者中静脉注射NBF006（一种带有siRNA的药物成分），研究的主要终点是药物的安全性、药代动力学及初步疗效（NCT03819387）。虽然这两种技术在操作上相对简单，基因沉默效果可靠^[67]^，但是它们的稳定性和靶向性较差，需要研究者们继续进行相关核酸化学修饰的研究。随着基因编辑技术的兴起，CRISPR（clustered regulary interspaced short palindromic repeats）的应用也越来越广泛，CRISPR/Cas9技术的发展促进了基因研究的进展，研究者通过CRISPR/dCas9-Tet1系统靶向去甲基化NNT CpG岛可显著降低其DNA甲基化水平，抑制A549/DDP细胞的DDP耐药性。基于CRISPR的DNA甲基化编辑NNT可能是治疗肺癌DDP耐药的潜在治疗方法^[56]^。CRISPR/dCas9的独特优势是它能够同时针对多个位点进行靶向的去甲基化，但是存在着一定的脱靶效应，提高识别效率将是该技术应用的关键^[68]^。

### 4.3 联合用药

由于肿瘤耐药是复杂、多机制的过程，因此联合治疗可能会获得更好的疗效。虽然研究显示DNMTs抑制剂对肺癌具有一定的疗效，但单独应用在肺癌治疗中仍然受限，根源在于疗效的有限性和较为严重的不良反应，如出现剂量限制性中性粒细胞减少症^[63]^。DNMT抑制剂联合其他药物会有更好的疗效。最常见的是DNMTs抑制剂联合标准抗肿瘤治疗，目前一项四氢尿酸-DAC联合帕博丽珠单抗治疗晚期或转移性NSCLC的临床研究正在进行中（NCT02664181）。另外，将DNMT抑制剂与组蛋白去乙酰化酶（histone deacetylases, HDAC）抑制剂联合使用也是一种有前景的治疗方案，两者可以发挥不同表观遗传学作用。在一项DNMTs抑制剂阿扎胞苷和HDAC抑制剂恩替诺特联合治疗复发转移性NSCLC患者的I期/II期试验^[69]^中，发现两者联用可以降低靶基因（RASSF1A、CDH13等）甲基化水平，增加后续化疗的疗效，延长肺癌患者的OS 。还有一项临床研究将DAC与丙戊酸联用，研究目的是确定其在NSCLC中的安全性和耐受性（NCT00084981），遗憾的是该项研究没有公布最终结果。

尽管既往的研究在逆转肺癌DDP耐药方面取得了一些进展，但其毒副作用依然突出，例如在一项试验^[69]^中，所有的患者均发生至少1例与治疗相关的不良事件，28%的患者出现了3级或4级不良反应，其中以疲劳和血液学毒性最常见，并且整个队列的中位生存期只有6.4个月。研究者们应针对现有的药物做进一步修饰以提高药物的安全性、稳定性和靶向性。还需要注意的是，与肺癌DDP耐药相关的DNA也有部分呈低甲基化，如前文提到的MRD1基因，因此仅针对基因的高甲基化研发药物是不足的，针对某些低甲基化基因研发促甲基化药物也是有前景的研究方向。同时非编码序列也应被给予更多关注。

## 5 小结与展望

在这个检测技术快速发展的时代，DNA甲基化检测因为无创、方便和快捷等优势受到广泛关注。随着研究的深入，研究者们利用DNA甲基化芯片对DNA甲基化谱进行分析，对耐药相关甲基化基因进行筛选。DNA甲基化在肺癌DDP耐药中的作用和机制愈发明确，这也为逆转肺癌DDP耐药提供了更多靶点和可能性。但是关于肺癌DDP耐药和DNA甲基化的研究才刚刚开始，还有很多问题需要解决。

首先，DNA甲基化检测的准确度和灵敏度仍然需要提高；其次，在细胞和小鼠实验中，针对大部分异常甲基化基因的研究仍然停留在表面，更深层的机制和信号通路尚未完全明确；最后，目前的临床试验结果并不令人满意，不良反应不易控制，这可能与逆转基因甲基化状态的具体机制仍不明确有关。因此，对于未来DNA甲基化与肺癌DDP耐药的研究可以从以下几个方面进行：（1）对已经筛选出来的靶基因进行下游分子信号通路的研究；（2）开展动物和临床试验，DNMTs抑制剂联合标准抗肿瘤治疗，观察其安全性和疗效；（3）开发新的、安全性更高的DNMTs抑制剂；（4）基因靶向治疗，将参与肺癌DDP耐药的基因作为靶点。肺癌DDP耐药由多因素、多分子共同介导，任何一种机制都不足以完全解释该现象的发生。未来不仅需要聚焦于DNA甲基化，同时也应关注与组蛋白乙酰化等其他表观遗传学的联合研究，从而挖掘出更深层的发生、作用机制，筛选出真正有效的靶点，找到逆转耐药的突破口。

## References

[1] Zhang Y, Luo G, Etxeberria J, et al. Global patterns and trends in lung cancer incidence: A population-based study. J Thorac Oncol, 2021, 16(6): 933-944. doi: 10.1016/j.jtho.2021.01.1626 33607309

[2] He S, Li H, Cao M, et al. Trends and risk factors of lung cancer in China. Chin J Cancer Res, 2020, 32(6): 683-694. doi: 10.21147/j.issn.1000-9604.2020.06.02 33446992 PMC7797233

[3] Johnson DH, Schiller JH, Bunn PA Jr. Recent clinical advances in lung cancer management. J Clin Oncol, 2014, 32(10): 973-982. doi: 10.1200/JCO.2013.53.1228 24567433

[4] Bodor JN, Kasireddy V, Borghaei H. First-line therapies for metastatic lung adenocarcinoma without a driver mutation. J Oncol Pract, 2018, 14(9): 529-535. doi: 10.1200/JOP.18.00250 30205771

[5] Zhang YW, Zheng Y, Wang JZ, et al. Integrated analysis of DNA methylation and mRNA expression profiling reveals candidate genes associated with cisplatin resistance in non-small cell lung cancer. Epigenetics, 2014, 9(6): 896-909. doi: 10.4161/epi.28601 24699858 PMC4065187

[6] Fu YS, Wang Q, Ma JX, et al. CRABP-II methylation: a critical determinant of retinoic acid resistance of medulloblastoma cells. Mol Oncol, 2012, 6(1): 48-61. doi: 10.1016/j.molonc.2011.11.004 22153617 PMC5528385

[7] Schiller JH, Harrington D, Belani CP, et al. Comparison of four chemotherapy regimens for advanced non-small-cell lung cancer. N Engl J Med, 2002, 346(2): 92-98. doi: 10.1056/NEJMoa011954 11784875

[8] Apetoh L, Ladoire S, Coukos G, et al. Combining immunotherapy and anticancer agents: the right path to achieve cancer cure? Ann Oncol, 2015, 26(9): 1813-1823. doi: 10.1093/annonc/mdv209 25922066

[9] Galluzzi L, Buqué A, Kepp O, et al. Immunological effects of conventional chemotherapy and targeted anticancer agents. Cancer Cell, 2015, 28(6): 690-714. doi: 10.1016/j.ccell.2015.10.012 26678337

[10] Aggarwal C, Borghaei H. Treatment paradigms for advanced non-small cell lung cancer at academic medical centers: involvement in clinical trial endpoint design. Oncologist, 2017, 22(6): 700-708. doi: 10.1634/theoncologist.2016-0345 28408617 PMC5469580

[11] Rabik CA, Dolan ME. Molecular mechanisms of resistance and toxicity associated with platinating agents. Cancer Treat Rev, 2007, 33(1): 9-23. doi: 10.1016/j.ctrv.2006.09.006 17084534 PMC1855222

[12] Teng JP, Yang ZY, Zhu YM, et al. Gemcitabine and cisplatin for treatment of lung cancer in vitro and vivo. Eur Rev Med Pharmacol Sci, 2018, 22(12): 3819-3825. doi: 10.26355/eurrev_201806_15266 29949158

[13] Gentilin E, Simoni E, Candito M, et al. Cisplatin-induced ototoxicity: updates on molecular targets. Trends Mol Med, 2019, 25(12): 1123-1132. doi: 10.1016/j.molmed.2019.08.002 31473143

[14] Fadejeva I, Olschewski H, Hrzenjak A. MicroRNAs as regulators of cisplatin-resistance in non-small cell lung carcinomas. Oncotarget, 2017, 8(70): 115754-115773. doi: 10.18632/oncotarget.22975 29383199 PMC5777811

[15] Lv P, Man S, Xie L, et al. Pathogenesis and therapeutic strategy in platinum resistance lung cancer. Biochim Biophys Acta Rev Cancer, 2021, 1876(1): 188577. doi: 10.1016/j.bbcan.2021.188577 34098035

[16] Qin T, Jelinek J, Si J, et al. Mechanisms of resistance to 5-aza-2’-deoxycytidine in human cancer cell lines. Blood, 2009, 113(3): 659-667. doi: 10.1182/blood-2008-02-140038 18931345 PMC2628372

[17] Bredberg A, Bodmer W. Cytostatic drug treatment causes seeding of gene promoter methylation. Eur J Cancer, 2007, 43(5): 947-954. doi: 10.1016/j.ejca.2006.12.003 17236756 PMC2430383

[18] Quintanal-Villalonga Á, Molina-Pinelo S. Epigenetics of lung cancer: a translational perspective. Cell Oncol (Dordr), 2019, 42(6): 739-756. doi: 10.1007/s13402-019-00465-9 31396859 PMC12994298

[19] Rajabi H, Tagde A, Alam M, et al. DNA methylation by DNMT1 and DNMT3b methyltransferases is driven by the MUC1-C oncoprotein in human carcinoma cells. Oncogene, 2016, 35(50): 6439-6445. doi: 10.1038/onc.2016.180 27212035 PMC5121097

[20] Zhang L, Dai Z, Yu J, et al. CpG-island-based annotation and analysis of human housekeeping genes. Brief Bioinform, 2021, 22(1): 515-525. doi: 10.1093/bib/bbz134 31982909

[21] Liu B, Song J, Luan J, et al. Promoter methylation status of tumor suppressor genes and inhibition of expression of DNA methyltransferase 1 in non-small cell lung cancer. Exp Biol Med (Maywood), 2016, 241(14): 1531-1539. doi: 10.1177/1535370216645211 27190263 PMC4994907

[22] Sandoval J, Heyn H, Moran S, et al. Validation of a DNA methylation microarray for 450,000 CpG sites in the human genome. Epigenetics, 2011, 6(6): 692-702. doi: 10.4161/epi.6.6.16196 21593595

[23] Shames DS, Minna JD, Gazdar AF. Methods for detecting DNA methylation in tumors: from bench to bedside. Cancer Lett, 2007, 251(2): 187-198. doi: 10.1016/j.canlet.2006.10.014 17166656

[24] Ashapkin VV, Kutueva LI, Vanyushin BF. Quantitative analysis of DNA methylation by bisulfite sequencing. Methods Mol Biol, 2020, 2138: 297-312. doi: 10.1007/978-1-0716-0471-7_21 32219758

[25] Vaisvila R, Ponnaluri VKC, Sun Z, et al. Enzymatic methyl sequencing detects DNA methylation at single-base resolution from picograms of DNA. Genome Res, 2021, 31(7): 1280-1289. doi: 10.1101/gr.266551.120 34140313 PMC8256858

[26] Sun Z, Vaisvila R, Hussong LM, et al. Nondestructive enzymatic deamination enables single-molecule long-read amplicon sequencing for the determination of 5-methylcytosine and 5-hydroxymethylcytosine at single-base resolution. Genome Res, 2021, 31(2): 291-300. doi: 10.1101/gr.265306.120 33468551 PMC7849414

[27] Liu Y, Hu Z, Cheng J, et al. Subtraction-free and bisulfite-free specific sequencing of 5-methylcytosine and its oxidized derivatives at base resolution. Nat Commun, 2021, 12(1): 618. doi: 10.1038/s41467-021-20920-2 33504799 PMC7840749

[28] Zhang J, Xie S, Xu J, et al. Cancer biomarkers discovery of methylation modification with direct high-throughput nanopore sequencing. Front Genet, 2021, 12: 672804. doi: 10.3389/fgene.2021.672804 34122526 PMC8188482

[29] Ardui S, Ameur A, Vermeesch JR, et al. Single molecule real-time (SMRT) sequencing comes of age: applications and utilities for medical diagnostics. Nucleic Acids Res, 2018, 46(5): 2159-2168. doi: 10.1093/nar/gky066 29401301 PMC5861413

[30] Yu WK, Wang Z, Fong CC, et al. Chemoresistant lung cancer stem cells display high DNA repair capability to remove cisplatin-induced DNA damage. Br J Pharmacol, 2017, 174(4): 302-313. doi: 10.1111/bph.13690 27933604 PMC5289946

[31] Martin LP, Hamilton TC, Schilder RJ. Platinum resistance: the role of DNA repair pathways. Clin Cancer Res, 2008, 14(5): 1291-1295. doi: 10.1158/1078-0432.CCR-07-2238 18316546

[32] Plumb JA, Steele N, Finn PW, et al. Epigenetic approaches to cancer therapy. Biochem Soc Trans, 2004, 32(Pt 6): 1095-1097. doi: 10.1042/BST0321095 15506976

[33] Kishi K, Doki Y, Yano M, et al. Reduced MLH1 expression after chemotherapy is an indicator for poor prognosis in esophageal cancers. Clin Cancer Res, 2003, 9(12): 4368-4375. 14555508

[34] Li A, Song J, Lai Q, et al. Hypermethylation of ATP-binding cassette B 1 (ABCB1) multidrug resistance 1 (MDR1) is associated with cisplatin resistance in the A549 lung adenocarcinoma cell line. Int J Exp Pathol, 2016, 97(6): 412-421. doi: 10.1111/iep.12212 27995666 PMC5370221

[35] Hanada S, Maeshima A, Matsuno Y, et al. Expression profile of early lung adenocarcinoma: identification of MRP 3 as a molecular marker for early progression. J Pathol, 2008, 216(1): 75-82. doi: 10.1002/path.2383 18604784

[36] Loe DW, Deeley RG, Cole SP. Biology of the multidrug resistance-associated protein, MRP. Eur J Cancer, 1996, 32A(6): 945-957. doi: 10.1016/0959- 8049(96)00046-9 10.1016/0959-8049(96)00046-9 8763335

[37] Nakayama M, Wada M, Harada T, et al. Hypomethylation status of CpG sites at the promoter region and overexpression of the human MDR1 gene in acute myeloid leukemias. Blood, 1998, 92(11): 4296-4307. 9834236

[38] Zhu Q, Center MS. Cloning and sequence analysis of the promoter region of the MRP gene of HL60 cells isolated for resistance to adriamycin. Cancer Res, 1994, 54(16): 4488-4492. 8044800

[39] Teng X, Wang SY, Shi YQ, et al. The role of emodin on cisplatin resistance reversal of lung adenocarcinoma A549/DDP cell. Anticancer Drugs, 2021, 32(9): 939-949. doi: 10.1097/CAD.0000000000001086 34001704

[40] Kong W, Ling X, Chen Y, et al. Hesperetin reverses P-glycoprotein-mediated cisplatin resistance in DDP-resistant human lung cancer cells via modulation of the nuclear factor-κB signaling pathway. Int J Mol Med, 2020, 45(4): 1213-1224. doi: 10.3892/ijmm.2020.4485 32124932 PMC7053858

[41] Malpeli G, Innamorati G, Decimo I, et al. Methylation dynamics of RASSF1A and its impact on cancer. Cancers (Basel), 2019, 11(7): 959. doi: 10.3390/cancers11070959 31323949 PMC6678546

[42] Mengxi D, Qian W, Nan W, et al. Effect of DNA methylation inhibitor on RASSF1A genes expression in non-small cell lung cancer cell line A549 and A549DDP. Cancer Cell Int, 2013, 13(1): 91. doi: 10.1186/1475-2867-13-91 24011511 PMC3846638

[43] Pfeifer GP, Rauch TA. DNA methylation patterns in lung carcinomas. Semin Cancer Biol, 2009, 19(3): 181-187. doi: 10.1016/j.semcancer.2009.02.008 19429482 PMC2680753

[44] Wang J, Zhang X, Yang F, et al. RASSF1A enhances chemosensitivity of NSCLC cells through activating autophagy by regulating MAP1S to inactivate Keap1-Nrf2 pathway. Drug Des Devel Ther, 2021, 15: 21-35. doi: 10.2147/DDDT.S269277 PMC7797300 33442234

[45] García-Gutiérrez L, McKenna S, Kolch W, et al. RASSF1A tumour suppressor: target the network for effective cancer therapy. Cancers (Basel), 2020, 12(1): 229. doi: 10.3390/cancers12010229 31963420 PMC7017281

[46] Zucchini C, Bianchini M, Valvassori L, et al. Identification of candidate genes involved in the reversal of malignant phenotype of osteosarcoma cells transfected with the liver/bone/kidney alkaline phosphatase gene. Bone, 2004, 34(4): 672-679. doi: 10.1016/j.bone.2003.12.008 15050898

[47] Feng Q, Hawes SE, Stern JE, et al. DNA methylation in tumor and matched normal tissues from non-small cell lung cancer patients. Cancer Epidemiol Biomarkers Prev, 2008, 17(3): 645-654. doi: 10.1158/1055-9965.EPI-07-2518 18349282 PMC2798850

[48] Wang Y, Zhang L, Yang J, et al. CDH13 promoter methylation regulates cisplatin resistance of non-small cell lung cancer cells. Oncol Lett, 2018, 16(5): 5715-5722. doi: 10.3892/ol.2018.9325 30344726 PMC6176259

[49] Mrkvicova A, Chmelarova M, Peterova E, et al. The effect of sodium butyrate and cisplatin on expression of EMT markers. PLoS One, 2019, 14(1): e0210889. doi: 10.1371/journal.pone.0210889 30653577 PMC6336326

[50] Böttger F, Semenova EA, Song JY, et al. Tumor heterogeneity underlies differential cisplatin sensitivity in mouse models of small-cell lung cancer. Cell Rep, 2019, 27(11): 3345-3358.e4. doi: 10.1016/j.celrep.2019.05.057 31189116 PMC6581744

[51] Cortés-Sempere M, de Miguel MP, Pernía O, et al. IGFBP-3 methylation-derived deficiency mediates the resistance to cisplatin through the activation of the IGFIR/Akt pathway in non-small cell lung cancer. Oncogene, 2013, 32(10): 1274-1283. doi: 10.1038/onc.2012.146 22543588

[52] Pernía O, Belda-Iniesta C, Pulido V, et al. Methylation status of IGFBP-3 as a useful clinical tool for deciding on a concomitant radiotherapy. Epigenetics, 2014, 9(11): 1446-1453. doi: 10.4161/15592294.2014.971626 25482372 PMC4622698

[53] Ibanez de Caceres I, Cortes-Sempere M, Moratilla C, et al. IGFBP-3 hypermethylation-derived deficiency mediates cisplatin resistance in non-small-cell lung cancer. Oncogene, 2010, 29(11): 1681-1690. doi: 10.1038/onc.2009.454 20023704

[54] Zhao J, Xue X, Fu W, et al. Epigenetic activation of FOXF 1 confers cancer stem cell properties to cisplatin-resistant non-small cell lung cancer. Int J Oncol, 2020, 56(5): 1083-1092. doi: 10.3892/ijo.2020.5003 32319573 PMC7115358

[55] Li N, Li X, Li S, et al. Cisplatin-induced downregulation of SOX1 increases drug resistance by activating autophagy in non-small cell lung cancer cell. Biochem Biophys Res Commun, 2013, 439(2): 187-190. doi: 10.1016/j.bbrc.2013.08.065 23994634

[56] Xu C, Jiang S, Ma X, et al. CRISPR-based DNA methylation editing of NNT rescues the cisplatin resistance of lung cancer cells by reducing autophagy. Arch Toxicol, 2023, 97(2): 441-456. doi: 10.1007/s00204-022-03404-0 36336710

[57] Zhang Y, Yuan Y, Li Y, et al. An inverse interaction between HOXA11 and HOXA11-AS is associated with cisplatin resistance in lung adenocarcinoma. Epigenetics, 2019, 14(10): 949-960. doi: 10.1080/15592294.2019.1625673 31144606 PMC6691981

[58] He T, Zhang M, Zheng R, et al. Methylation of SLFN11 is a marker of poor prognosis and cisplatin resistance in colorectal cancer. Epigenomics, 2017, 9(6): 849-862. doi: 10.2217/epi-2017-0019 28403629

[59] Nogales V, Reinhold WC, Varma S, et al. Epigenetic inactivation of the putative DNA/RNA helicase SLFN11 in human cancer confers resistance to platinum drugs. Oncotarget, 2016, 7(3): 3084-3097. doi: 10.18632/oncotarget.6413 26625211 PMC4823092

[60] Tian FM, Zhong CY, Wang XN, et al. PDE3A is hypermethylated in cisplatin resistant non-small cell lung cancer cells and is a modulator of chemotherapy response. Eur Rev Med Pharmacol Sci, 2017, 21(11): 2635-2641. 28678321

[61] Kim D, Kim DH. Epigenome-based precision medicine in lung cancer. Methods Mol Biol, 2018, 1856: 57-85. doi: 10.1007/978-1-4939-8751-1_4 30178246

[62] Kantarjian H, Oki Y, Garcia-Manero G, et al. Results of a randomized study of 3 schedules of low-dose decitabine in higher-risk myelodysplastic syndrome and chronic myelomonocytic leukemia. Blood, 2007, 109(1): 52-57. doi: 10.1182/blood-2006-05-021162 16882708

[63] Schrump DS, Fischette MR, Nguyen DM, et al. Phase I study of decitabine-mediated gene expression in patients with cancers involving the lungs, esophagus, or pleura. Clin Cancer Res, 2006, 12(19): 5777-5785. doi: 10.1158/1078-0432.CCR-06-0669 17020984

[64] Duan L, Perez RE, Chastain PD 2nd, et al. JMJD 2 promotes acquired cisplatin resistance in non-small cell lung carcinoma cells. Oncogene, 2019, 38(28): 5643-5657. doi: 10.1038/s41388-019-0814-6 30967636

[65] Jorgensen R. Altered gene expression in plants due to trans interactions between homologous genes. Trends Biotechnol, 1990, 8(12): 340-344. doi: 10.1016/0167-7799(90)90220-r 1366894

[66] Elbashir SM, Harborth J, Lendeckel W, et al. Duplexes of 21-nucleotide RNAs mediate RNA interference in cultured mammalian cells. Nature, 2001, 411(6836): 494-498. doi: 10.1038/35078107 11373684

[67] Mamidipalli S, Palakal M, Li S. OligoMatcher: analysis and selection of specific oligonucleotide sequences for gene silencing by antisense or siRNA. Appl Bioinformatics, 2006, 5(2): 121-124. doi: 10.2165/00822942-200605020-00008 16722778

[68] Boyle EA, Andreasson JOL, Chircus LM, et al. High-throughput biochemical profiling reveals sequence determinants of dCas 9 off-target binding and unbinding. Proc Natl Acad Sci U S A, 2017, 114(21): 5461-5466. doi: 10.1073/pnas.1700557114 28495970 PMC5448226

[69] Juergens RA, Wrangle J, Vendetti FP, et al. Combination epigenetic therapy has efficacy in patients with refractory advanced non-small cell lung cancer. Cancer Discov, 2011, 1(7): 598-607. doi: 10.1158/2159-8290.CD-11-0214 22586682 10.1158/2159-8290.CD-11-0214 PMC3353724

